# Active thoracic kyphotisation: modifications in the shape of the trunk

**DOI:** 10.1186/1748-7161-8-S1-O23

**Published:** 2013-06-03

**Authors:** M Romano, F Saveri, A Negrini, S Negrini

**Affiliations:** 1 ISICO (Italian Scientific Spine Institute), Milan, Italy; 2 University of Brescia, Brescia, Italy; 3 IRCCS Don Gnocchi, Milan, Italy

## Background

The three-dimensional development of scoliosis has always led the experts in this field to say that the correction of curves in the sagittal plane is an important topic. Many experts propose that the thoracic correction should be done very carefully, to avoid any increase of kyphosis resulting in an increase of the hump.

## Aim

The primary purpose of this study is to verify the changes of the shape of the trunk in subjects, when asked to actively increase their thoracic kyphosis. Secondary purpose was to investigate the differences in the results while the subjects inhaled, or exhaled.

## Method

37 consecutive patients (28 females - 9 males; Average age 14.9±2.3; Average °Cobb 27.05±12.9) performed 4 different postural examinations with a Formetric system in orthostatic position.

(1) in the normal position (NP)

(1) in a position of Active Self-Correction (ASC)

(1) kyphotisation in the inspiratory phase (KI)

(1) kyphotisation in the expiratory phase (KE)

## Results

In NP, the average measure of thoracic kyphosis was 37.8°±11.2. In comparison to the normal position, the extent of thoracic kyphosis actually increases when the patient tries actively to emphasize it, both during an inspiratory (43.7±10.4), and an expiratory (45.6±39.7), phase. The average area of the hump doesn't change significantly: 6.4±3.5 in NP, 6.5±3.5 in SC, 6.5±3.0 during KI, 6.5±3.1 during KE. The pattern of modification of the hump area is unclear. In 23% of subjects, we find the highest hump in NP, in 28% of patients in SC, in 28% during KI, and 21% during KE. The average value of lumbar plumbline distance (PD) is 40.3 in NP, 27.0 in SC, 61,4 during KI and 63.4 during KE. The average value of cervical PD is 29.4 in NP, 24.8 in SC, 27,9 during KI and 28.9 during KE.

## Conclusions

In orthostatic position, it's possible to actively increase the thoracic kyphosis. Performing this movement in inhalation, or exhalation, can help the subject, but the pattern is not the same for all subjects. Area of the hump changes differently, depending on the subject. In lumbar curves, we find a reduction of cervical PD during ASC, and an increase value both in KI than in KE.
